# Inorganic Nanoparticle-Loaded Exosomes for Biomedical Applications

**DOI:** 10.3390/molecules26041135

**Published:** 2021-02-20

**Authors:** Taraneh Barjesteh, Shomit Mansur, Yuping Bao

**Affiliations:** Department of Chemical and Biological Engineering, The University of Alabama, Box 870203, Tuscaloosa, AL 35487, USA; tbarjesteh@crimson.ua.edu (T.B.); smansur@crimson.ua.edu (S.M.)

**Keywords:** exosomes, inorganic nanoparticles, bioimaging, biomimetic nanocarriers, theranostics

## Abstract

Exosomes are intrinsic cell-derived membrane vesicles in the size range of 40–100 nm, serving as great biomimetic nanocarriers for biomedical applications. These nanocarriers are known to bypass biological barriers, such as the blood–brain barrier, with great potential in treating brain diseases. Exosomes are also shown to be closely associated with cancer metastasis, making them great candidates for tumor targeting. However, the clinical translation of exosomes are facing certain critical challenges, such as reproducible production and *in vivo* tracking of their localization, distribution, and ultimate fate. Recently, inorganic nanoparticle-loaded exosomes have been shown great benefits in addressing these issues. In this review article, we will discuss the preparation methods of inorganic nanoparticle-loaded exosomes, and their applications in bioimaging and therapy. In addition, we will briefly discuss their potentials in exosome purification.

## 1. Introduction

Exosomes are intrinsic cell-derived membrane vesicles in a size range of 40–100 nm, and are naturally secreted by different cells [1,2]. Typically, exosomes contain biological components from the parental cells, such as mRNA, transporting proteins, or proteins associated with specific functions of the secreting cells [1,2]. Because of the contents of signaling molecules, optimal sizes, and membrane coatings, exosomes are known to have the ability to bypass biological barriers, including the blood–brain barrier, serving as powerful drug and gene therapy transporters [3]. Exosomes are also known to enhance cell proliferation [4], and are closely associated with cancer metastasis [5,6]; thus, tumor cell-derived exosomes intrinsically exhibited tumor targeting ability. Therefore, exosomes have been extensively studied as nanocarriers for drug delivery [7,8,9], where the cargos can be either introduced by treating parental cells with cargos of interests or loading cargos into exosomes post exosome isolation [10]. The potential benefits of exosomes have been demonstrated in various applications, such as cancer metastasis [11,12], musculoskeletal disorders [13], and nerve repair [14,15].

Despite the great potentials of exosomes, two critical challenges in exosome field remain: reproducible preparation and effective *in vivo* exosome tracking for therapeutic applications. Even though several techniques have been developed for tracking exosomes [16], limitations remain depending on the imaging modalities. For example, optical imaging by labeling exosomes with fluorescence molecules are widely utilized [16]. Fluorescence imaging is a highly sensitive and cost-effective imaging technique, but it suffers from deep tissue penetration, and background interferences. Positron emission tomography (PET) is a sensitive *in vivo* imaging technique, but it has limited usage in long-term tracking because of the short lifetime of the radioactive tracers. Inorganic nanoparticles provide many advantages as imaging probes, such as highly reproducible preparation and functionalization methods, long-term tracking ability, and potential of multifunctionality. For instance, iron oxide nanoparticles have been long studied as contrast agents for magnetic resonance imaging (MRI) [17]. In contrast, gold (Au) nanoparticles have been explored as effective contrast agents for computerized tomography (CT) [18]. In addition, inorganic nanoparticles can serve as a means to deliver therapeutic effects, such as Au nanocages for photothermal therapy [19]. Therefore, loading exosomes with inorganic nanoparticles provides a great strategy for *in vivo* tracking and therapy. In addition, it was recently demonstrated that iron oxide nanoparticles with rationally designed surface coatings were used for effective removal of other biological components, assisting exosome purification [20].

A number of reviews have been available on the preparation, isolation, and biological functions of exosomes [21,22,23,24]. This review specifically discusses the role of inorganic nanoparticles in inorganic nanoparticle/exosome hybrid nanoconstructs, including methods to incorporate inorganic nanoparticles to exosomes and their specific roles in bioimaging, exosome tracking, and theranostics.

## 2. Preparation of Nanoparticle-Loaded Exosomes

**Figure 1:**
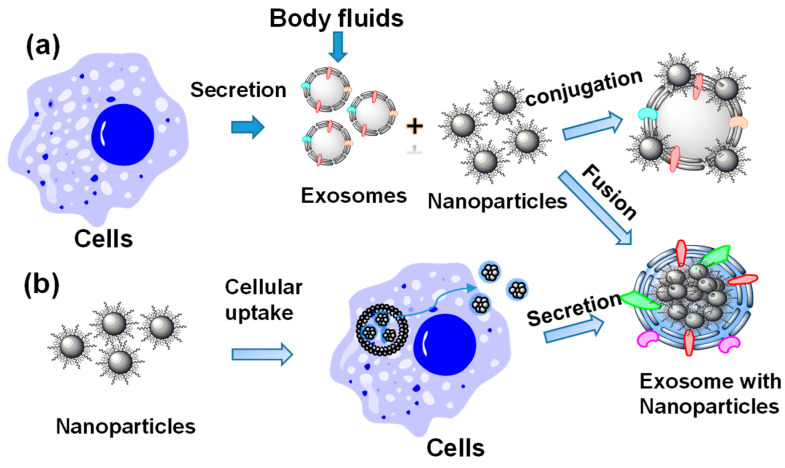
A schematic of preparation methods of inorganic nanoparticle-loaded exosomes: (**a**) loading nanoparticles post-exosome formation by either surface conjugation or fusion, (**b**) loading nanoparticles during exome-formation inside the cells by treating cells with nanoparticles.

Exosomes can be integrated with inorganic nanoparticles via several approaches, either post exosome formation or during exosome formation [23], as shown in Figure 1. Exosomes are naturally present in body fluids, and can be secreted by different types of cells. These exosomes can be isolated and then incubated with inorganic nanoparticles to form hybrid nanostructures. On the other hand, nanoparticles can be introduced into exosome-secreting cells via cellular uptake and are subsequently simultaneously incorporated into exosomes during secretion.

### 2.1. Preparation of Nanoparticle-Loaded Exosomes Post-Exosome Formation

Nanoparticle-loaded exosomes can be prepared after exosome isolation from cell culture or body fluids, where the nanoparticle loading can be achieved by direct surface conjugation or fusion via electroporation, extrusion, thermal shock, or sonication [22].

Direct surface conjugation was mainly achieved by attaching chemical linkers to exosome surfaces via covalent binding to surface proteins [25] or hydrophobic tail insertion of molecules with click chemistry to lipid bilayer of exosomes [26]. These functional linkers subsequently reacted with the functional surfaces of nanoparticles, leading to a “raspberry” nanoconstruct where the exosomes were decorated with inorganic nanoparticles.

**Figure 2:**
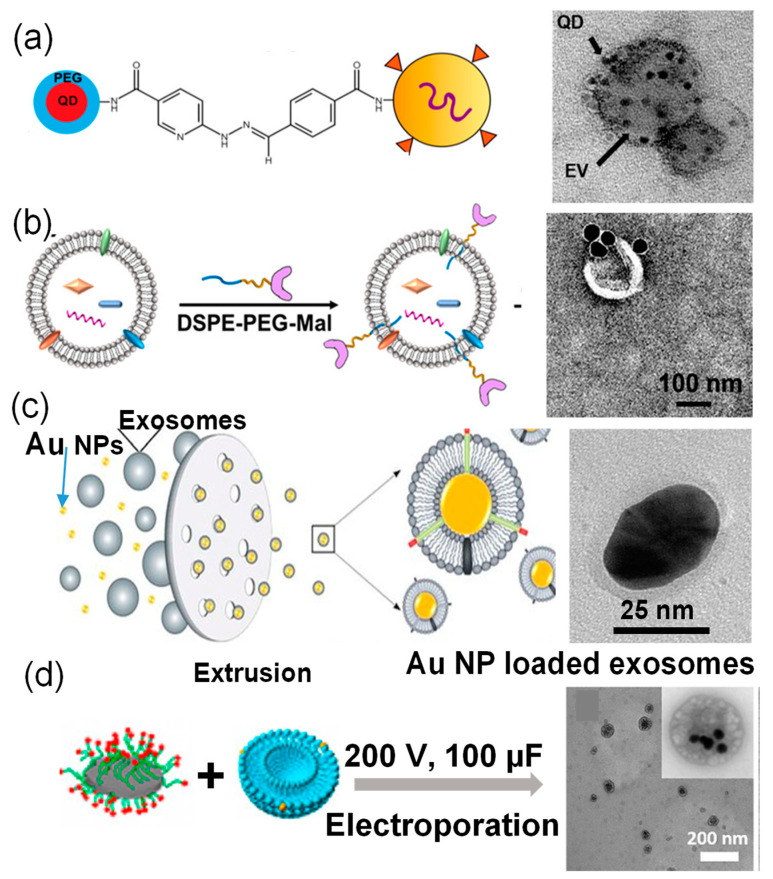
Examples of inorganic nanoparticle (NP)-loaded exosomes post exosome formation with schematic drawings and TEM images: (**a**) QD-loaded exosomes through chemical linker conjugation, [25] (**b**) Au NP-loaded exosomes through lipid infused linkers, [26] (**c**) Au NP-loaded exosomes through extrusion, [28] and (**d**) V_2_C DD-loaded exosomes via electroporation [30].

For example, a hydrazine–aldehyde-based linking strategy based on 4-formylbenzoate (4FB) to 6-hydrazinonicotinate acetone hydrazone (HyNic) click chemistry was explored to label exosomes with quantum dots (QDs) [25]. Specifically, commercially available 10–20 nm QDs functionalized with amine-terminated polyethylene glycol (QD-PEG-NH_2_) were modified with Sulfo-S-4FB, while exosomes isolated from body fluids were labeled with Sulfo-S-HyNic through the interactions between amine-reactive NHS-esters and exosome surface proteins. The successful conjugation was confirmed by transmission electron microscopy (TEM) images, where QDs were decorated on the surface of exosomes (Figure 2a). Because of differences in electron density, QDs appeared much darker than exosomes. The co-localization of QDs and exosomes was also confirmed with the overlapping fluorescence signals of fluorescence-stained exosomes and the QDs. In addition to linker conjugation, linkers can be displayed on exosome surfaces through linkers with a hydrophobic tail, which can be directly inserted into the membrane of exosomes. For example, a hydrophobic insertion strategy was applied to insert maleimide-terminated 1,2-Distearoyl-sn-glycero-3-phosphoethanolamine (DSPE)-PEG linker with a hydrophobic chain (DSPE), a hydrophilic linker (PEG), and a Mal reactive end (Figure 2b) [26]. DSPE is able to insert into membrane structures spontaneously through hydrophobic interactions, while PEG possesses good biocompatibility and hydrophilicity for improved solubility. The Mal group was used to conjugate thiol-bearing nanoparticles via high-efficiency click chemistry. Subsequently, 40 nm Au nanoparticles functionalized with thiol groups were effectively attached to the surfaces of MCF-7 cell derived exosomes (Figure 2b). The surface conjugation strategies using chemical linkers to attach inorganic nanoparticles to exosome surfaces must be carefully selected [25] to minimize the disruption of exosome structural integrity. In addition, the water solubility of the linker is critical, because the use of organic solvents would cause potential damage to exosomes. This technique mainly leads to the attachment of inorganic nanoparticles to exosome surfaces rather than inside the exosomes.

Alternatively, inorganic nanoparticles can be loaded inside exosomes post exosome formation [27]. Because of the low loading efficiency of passive loading by simply mixing nanoparticles and exosomes, physical alternation is normally applied to infuse inorganic nanoparticles inside exosomes, such as electroporation, extrusion, sonication, and freeze–thaw [27]. These physical approaches cause membrane rearrangements of exosomes or temporal opening, allowing for nanoparticle inclusion. For example, Au nanoparticles were successfully coated with exosomes derived from transformed human embryonic kidneys (HEK293T) cells through a serial extrusion method [28]. During preparation, the mixture of 20–30 nm Au nanoparticles and purified exosomes from was simply incubated in phosphate buffered saline (PBS) at 4 °C for 30 min, followed by serial extrusion through 400 nm, 200 nm, and 100 nm polycarbonate porous membranes. This extrusion process led to a good exosome coating on the Au nanoparticle surface (Figure 2c). However, the yield of the nanoparticle-loaded exosomes was rather low, likely due to the adsorption of nanoparticles and exosomes to the porous membrane. Electroporation is often utilized to transfer cargos across cellular membranes by applying a voltage potential from one side of a cell membrane to the other for spontaneous pore formation. This technique has been applied to load cargos to exosomes, including inorganic nanoparticles [29]. Figure 2d shows an example of loading 16 nm vanadium carbide quantum dots (V_2_C QDs) into exosomes derived from breast cancer (MCF-7) cells via electroporation (200 V and 100 µF) [30]. Electroporation minimizes the disruption of delicate components associated exosomes, such as receptors, but the elevated potential could cause exosome aggregation. Therefore, in most cases, a liposome stabilizer (e.g., trehalose) is introduced into the pulse media. For example, 5 nm of superparamagnetic iron oxide nanoparticles was successfully loaded into exosomes isolated from melanoma B16-F10 cells in the presence of 50 mM trehalose in PBS [29]. Loading 5 nm nanoparticles with electroporation did not alter the original size of exosomes. A similar strategy was applied to load hollow Au nanoparticles into exosomes derived from B16-F10 cells, but the loading efficiency was really low (<10%) and significant alternation of exosome morphology was observed [27]. The low loading efficiency and morphological alternation likely resulted from the large size of hollow Au nanoparticles (~40 nm). In another study, near infrared (NIR) quantum dots, silver selenium (Ag_2_Se), were loaded into exosome derived from cancer cells through electroporation. The nanoparticle loading efficiency was reported to be over 90%, because of the extremely small sizes of those QDs (~2 nm) [31]. This study further indicated the importance of the nanoparticle size in creating nanoparticle-loaded exosomes. Sonication of nanoparticle and exosome mixture is another common strategy to load nanoparticles into exosomes. During this process, the exosome membrane integrity is disrupted by the mechanical shear force of the sonication source, which allows nanoparticles to fuse through the disrupted membrane. This process is highly dependent on the experimental conditions, and the heat generated from the sonication could cause damage to the exosomes [27]. Using hollow Au nanoparticles as a model system, the cross-comparison among different strategies suggested a similar nanoparticle loading efficiency among those methods, but nanoparticle loading via biological pathway was significantly higher [27].

In addition to surface conjugation and physical approaches, glucose-coated Au nanoparticles were shown to be taken up by exosomes isolated from mesenchymal stem cells [32]. The uptake mechanism was attributed to the glucose coatings of Au nanoparticles through GLUT-1 glucose transporters in exosome membranes. These studies suggested that regardless of the preparation methods, the size and surface functionalization of inorganic nanoparticles are critical for the successful formation of nanoparticle-loaded exosomes. A summary of inorganic nanoparticle-loaded exosomes through physical methods is shown in Table 1, where the experimental conditions were only reported by certain studies.

### 2.2. Preparation of Nanoparticle-Loaded Exosomes during Exosome Formation through Biological Pathways

Nanoparticle loading via biological pathways has been a primary method of efficiently loading nanoparticles inside exosomes [27,33]. During this process, exosome-secreting cells are incubated with surface functionalized nanoparticles for a certain period of time (normally 24 h or 48 h), allowing nanoparticles to be internalized by cells mainly via endocytosis [33]. During exosome formation, the internalized nanoparticles by cells are sponanusely incoporated into the secreting exosomes. To enhance the yield of exosomes, the nanoparticle-treated cells are normally cultured for an additional period of time under stressed culturing conditions, such as depletion of serum supplement, hypoxic media, or under radiation [34]. For example, iron oxide nanoparticles were efficiently loaded into exosomes derived from metastatic breast cancer, MDA-MB-231, cells. During this process, radiation and hypoxia were shown to increase the number of exosomes, and the highest yield was observed for the combination of radiation and hypoxic conditions. In addition, the preparation conditions of the exosomes affected the exosome targeting abilities where exosomes produced at hypoxic conditions were shown to be preferentially taken up by hypoxic cells [34]. A compassion study using hollow Au nanoparticles has shown that nanoparticle treatment of cells led to over 50% of nanoparticle loading efficiency, which was significantly higher than other methods (<20%) [27].

**Figure 3:**
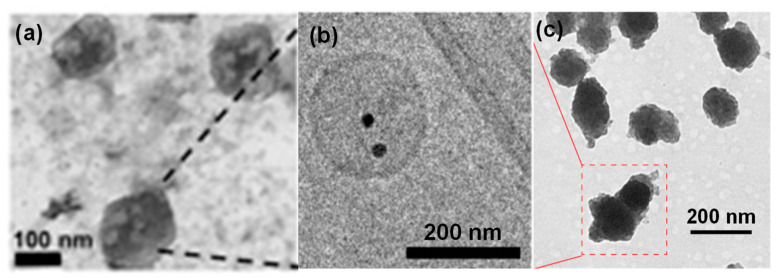
TEM images of some typical examples of inorganic nanoparticle-loaded exosomes: (**a**) iron oxide nanoparticles-loaded exosomes derived from MSCs [35], (**b**) Au nanoparticle-loaded exosomes derived from melanoma B16F10 cells [36], and (**c**) porous silicon nanoparticles-loaded exosomes derived from Bel7402 cells [37].

Figure 3 shows the TEM images of some typical examples of inorganic nanoparticle-loaded exosomes prepared through biological pathways. Figure 3a shows iron oxide nanoparticle-loaded exosomes derived from MDA-MB-231 cells, where PEG-functionalized iron oxide nanoparticles (20–30 nm) were successfully encapsulated inside exosomes by culturing nanoparticles treated mesenchymal stem cells (MSCs) for 24 h [35]. In another example, citrate-coated 12 nm Au nanoparticles modified with polyethylene glycol and folic acid were successfully incorporated in exosomes derived from melanoma B16F10 cells for caner targeting and *in vivo* tracking (Figure 3b) [36]. Porous silicon or drug loaded porous silicon nanoparticles were coated with exosomes secreted by human hepatocarcinoma Bel7402 cells for efficient lung cancer chemotherapy [37], as shown in Figure 3c.

For nanoparticle-loaded exosomes produced via biological pathways, the nanoparticle parameters (e.g., size and surface coatings) are critical to cellular uptake and subsequent exosome formation. In addition, the size distribution and morphology of nanoparticle-loaded exosomes are not as well controlled as those produced by methods post exosome formation. In addition to nanoparticle treatment, it has been shown that inorganic nanoparticles can be synthesized by cells in situ, leading to nanoparticle-loaded exosomes. For example, iron oxide and Ag hybrid nanoparticles were synthesized by incubating liver HepG2 cells with iron and silver inorganic salts, which directly resulted in hybrid nanoparticles loaded exosomes. These Ag-Fe_3_O_4_ nanoclusters loaded exosomes exhibited potential as both CT and MRI contrast agents for cancer diagnosis [38].

## 3. Applications of Inorganic Nanoparticle-Loaded Exosomes

Inorganic nanoparticle-loaded exosomes have been explored for numerous applications, including *in vivo* tracking, simultaneous imaging and therapy, bio-distribution analysis, etc. [39,40]. The inorganic nanoparticles are normally used as contrast agents or imaging probes for certain imaging modalities, such as iron oxide nanoparticles for MRI and MPI, Au nanoparticles for CT imaging, QDs for fluorescence imaging, or Au/Ag nanoparticles for surface enhanced Raman spectrometry (SERS) detection. Additionally, these inorganic nanoparticles can serve as a means to deliver therapy, such as magnetic nanoparticles for magnetic hypothermia or nanoparticles with NIR absorbance for photothermal therapy. This section will mainly discuss the potential of nanoparticle-loaded exosomes in *in vivo* tracking using different imaging modalities and therapy.

### 3.1. Inorganic Nanoparticle-Loaded Exosomes for Bioimaging

For inorganic nanoparticle-loaded exosomes, the inorganic nanoparticles were primarily used as contrast agents or imaging probes to track exomes in targeting, trafficking, or distributions [17,29]. Among different imaging modalities, MRI and CT imaging are the most attractive because of their imaging resolution, well-studied inorganic nanoparticle contrast agents, and noninvasive nature of the techniques. Even though QDs have great potential in fluorescence imaging, their *in vivo* applications have been limited by the deep tissue penetration and background interferences.

**Figure 4:**
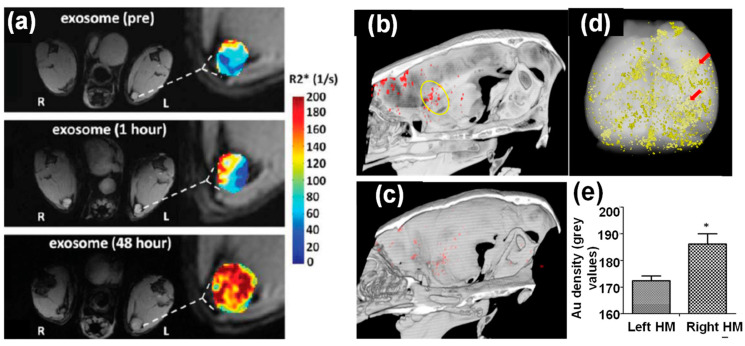
(**a**) *In vivo* MR imaging of mice injected with iron oxide nanoparticle-loaded exosomes derived from melanoma cells. After 48 h footpad injection, the _T1_-weighted images and R2* mapping clearly suggested accumulation of exosomes in lymph node [42]. (**b**–**e**) *In vivo* CT imaging of Au nanoparticle-loaded exosomes after intranasal administration into mice with acute striatal stroke: (**b**) a CT image 24 h post-administration (the ischemic region is indicated by the yellow circle), (**c**) a CT image of control animal with saline injection, (**d**) *ex vivo* CT imaging and Au quantification within the animal brain 24 h post administration, and (**e**) CT analysis of Au density of left and right hemispheres (note: stroke was introduced in the right hemisphere) [32].*: (*p* < 0.05).

As a noninvasive and non-ionizing imaging technique, MRI requires contrast agents for enhanced imaging resolution where iron oxide nanoparticles serve as a great option [41]. Even though gadolinium-based complexes are clinical options as MRI contrast agents, loading magnetic nanoparticles within exosomes have been primarily studied for exosome imaging because of their high relaxivity and easy functionalization [41]. For example, 5 nm iron oxide nanoparticles loaded exosomes derived from B16-F10 cells were used to track the localization of exosomes in lymph nodes [42]. These iron oxide nanoparticle-loaded exosomes were introduced into mice via footpad injection. After 48 h, preferential accumulation of those exosomes was observed at the resident structural regions of the lymph node, indicated by significant signal enhancement of the _T1_-weighted MR images (Figure 4a). The effectivity of using MR imaging to track iron oxide nanoparticle-loaded exosomes was also demonstrated by adipose stem cell-derived vesicles loaded with ultrasmall iron oxide nanoparticles [43]. *In vivo* MR images clearly detected those iron oxide nanoparticle-loaded exosomes in the muscular tissue after intramuscular injection. In addition to serving as MRI contrast agents, magnetic iron oxide nanoparticles are also great tracers for magnetic particle imaging (MPI), an emerging imaging modality for *in vivo* tomographic imaging with minimal background interference [44]. MPI has been used to track iron oxide nanoparticle-loaded exosomes derived from metastatic breast cancer, MDA-MB231 cells. Those cancer cell-derived exosomes were effectively accumulated at tumor sites and were taken up by hypoxic cancer cells [34].

CT imaging is a cost-effective, highly accessible, and efficient imaging modality with high temporal and spatial resolution. In addition to clinically used iodine-based compounds, Au nanoparticles have been explored extensively as CT contrast agents because of their high imaging resolution and demonstrated safety in preclinical studies [18]. Therefore, Au nanoparticle-loaded exosomes can be effectively tracked by CT imaging *in vivo*. For example, bone marrow MSC-derived exosomes labeled with glucose-coated Au nanoparticles were introduced into mice with acute striatal stroke via intranasal administration. These exosomes showed preferential accumulation inside the brain compared to intravenous injection. In addition, *in vivo* CT imaging suggested elective localization of these exosomes at stroke region 24 h post injection (Figure 4b–e), which was further verified by fluorescence imaging using a fluorescent marker [32]. CT imaging using the same type of Au nanoparticle-loaded exosomes further demonstrated their great potential in targeting and accumulating at different brain pathologies, including stroke, autism, Parkinson’s disease, and Alzheimer’s disease using pathologically relevant murine models up to 96 h post administration [45]. In contrast, healthy control animals showed a diffuse migration pattern and clearance of these exosomes by 24 h. The targeting mechanism of these exosomes was hypothesized to be inflammatory-driven, where MSC derived exosomes were selectively taken up by neuronal cells, but not glial cells, in the pathological regions [45]. The ability of those Au nanoparticle-loaded exosomes penetrating the BBB after injection was key to the brain accumulation.

**Figure 5:**
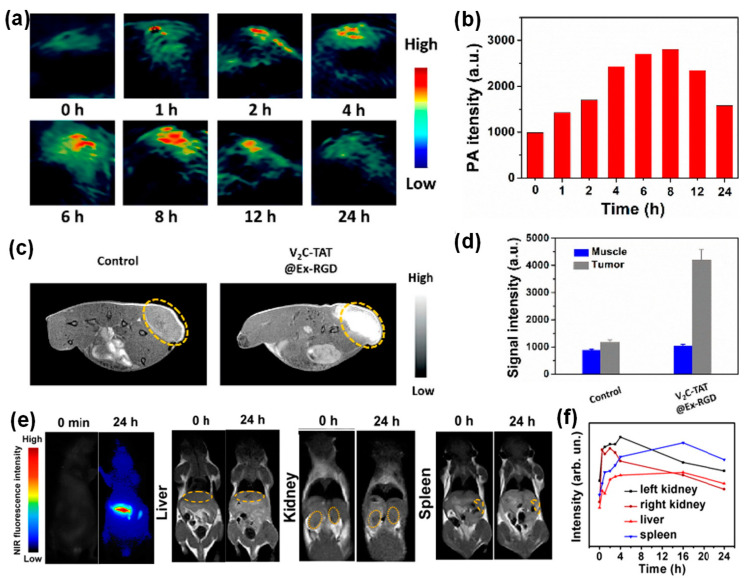
Dual imaging modalities with inorganic nanoparticle-loaded exosomes: (**a**–**d**) *in vivo* imaging of MCF-7 breast tumor-bearing mice after intravenous injection of V_2_C QD-loaded exosomes (**a**,**b**) PA images and quantified PA intensities of tumor sites at different times, (**c**,**d**) _T1_-weighted MR images and quantified MR signals of tumor sites 24 h post injection [30]. (**e**) NIR fluorescence and _T1_-weighted images of mouse liver, kidneys, and spleen at 0 h and 24 h post injection of Ag_2_Se@Mn QD-labeled exosomes. (**f**) MR signal quantification in mouse liver, kidneys, and spleen after injection of Ag_2_Se@Mn QD-labeled exosomes [31].

Another major advantage of inorganic nanoparticles is easy engineering for multi-functionality, allowing for dual mode tracking or simultaneous imaging and therapy. For example, cell-derived vesicles loaded with Au-iron oxide nanoparticles enabled both NIR fluorescence and MR imaging [46]. In another example, V_2_C QDs were successfully loaded into exosomes derived from MCF-7 breast cancer cells via electroporation [30]. Here, the QDs were modified with TAT peptides for nucleus targeting and the exosomes were functionalized with Arg-Gly-Asp (RGD) peptide for cell targeting. These exosomes exhibited distinct NIR absorbance from V2C QDs, serving as great contrast agents for photoacoustic imaging (PAI). PAI uses a radio frequency-pulsed laser to irritate the region of interest, and the thermal expansion caused by the absorbed heat leads to acoustic wave detectable by ultrasounds for an image [30]. Figure 5a showed *in vivo* PA images of MCF-7 breast tumor-bearing mice at different times post injection of these V_2_C QD-loaded and functionalized exosomes. The PA signal reached to a maximum around 8 h at the tumor site. The PA signal quantification agreed well with the imaging observation (Figure 5d). Additionally, V_2_C QDs can serve as MRI contrast agents due to the 3D electronic configuration of V^4+^ and quantum confinement, leading to multi-modal imaging. Figure 5c shows the _T1_-weighted MR images of MCF-7 breast tumor-bearing mice 24 h post-exosome injection. Significant MR signal enhancement was observed compared to the control mice with saline injection, which was consistent with the MR signal quantification results (Figure 5d).

In another study, NIR QDs, silver selenium (Ag_2_Se), were magnetically functionalized with manganese ion (Mn^2+^), leading to magnetic and fluorescent bifunctional nanoparticles [31]. Subsequently, these nanoparticles were introduced into exosomes derived from several types of cancer cells, such as human oral squamous cell carcinoma (CAL27) cells, human lung epithelial carcinoma (A549) cells, human breast cancer (MCF-7) cells, and human immortalized noncancerous keratinocytes (HaCaT) cells through electroporation. It was shown that the nanoparticle loading efficiency was not affected by the exosome secreting cell types and the extremely size of these QDs led to over 90% loading efficiency. These magnetic Ag_2_Se-labeled exosomes enabled dual-mode tracking with fluorescence and MRI [31]. Figure 5e shows the NIR fluorescence and _T1_-weighted images of mouse liver, kidneys, and spleen at 0 h and 24 h post injection of Ag_2_Se@Mn QD-labeled exosomes. The NIR images showed significant signal enhancement compared to the control, while the MR signal enhancement was not profound likely do to the low density of manganese on the QD surfaces. The MR signal quantification at those organs after injection of the Ag_2_Se@Mn QD-labeled exosomes was in line with the imaging observation in Figure 5f.

In addition to *in vivo* tracking, inorganic nanoparticle-loaded exosomes were explored for cellular sensing. For example, Au nanoparticle-loaded exosomes derived from Hela cells were shown to be SERS active. The cellular uptake and intracellular fates of those can be monitored by SERS [47].

### 3.2. Inorganic Nanoparticle Loaded Exosomes for Theranostic Applications

Besides exosome tracking with different imaging modalities, inorganic nanoparticle-loaded exosomes have been explored as a tool for simultaneous imaging and therapy [23]. To this end, the therapeutic function was achieved by either encapsulating therapeutic molecules into the inorganic nanoparticles [37] or using inorganic nanoparticles directly as a means to induce therapy, such as photothermal therapy [30]. For example, doxorubicin (DOX)-loaded silicon porous nanoparticles were successfully loaded inside exosomes derived from human hepatocarcinoma Bel7402 cells. The intrinsic fluorescent properties of silicon allowed for exosome tracking with fluorescence imaging and DOX molecules trapped inside silicon pores delivered therapeutic effects. These tumor cell-derived exosomes showed enhanced tumor accumulation with deep tumor penetration after being injected into tumor-bearing mice [37]. The antitumor activities of these exosomes were demonstrated in subcutaneous transplantation tumor model, orthotropic tumor model, and advanced metastatic tumor model, where the anticancer ability of these exosomes was attributed to the cancer stem cell targeting and killing.

**Figure 6:**
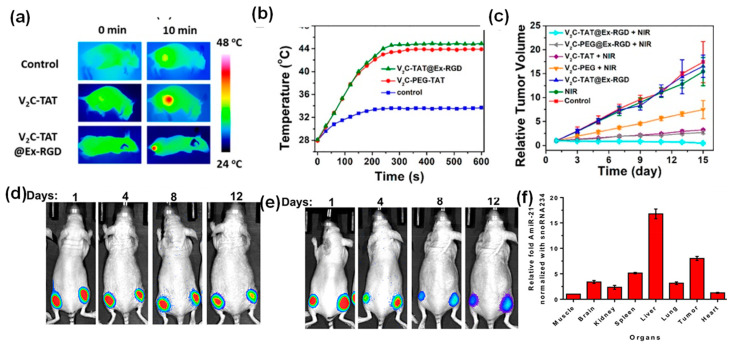
(**a**) IR thermal images and (**b**) the corresponding temperature changes at the tumor sites of MCF-7 tumor-bearing mice under 1064 nm laser irradiation. (**c**) Relative tumor growth curves of the MCF-7 tumor-bearing mice in different groups [30]. (**d**–**f**) *In vivo* bioluminescence imaging for the 14-day growth of xenografts of 4_T1_-Fluc-eGFP cells implanted in nude mice post administration of Au-Iron oxide nanoparticle-loaded exosomes. (**d**–**f**) *In vivo* biodistribution and anti-tumor properties of tumor cell derived exosomes loaded with Au-iron oxide nanoparticles and anti-miR-21 in combination with DOX. The bioluminescence images of animals treated with exosomes without DOX (**d**) and with DOX (**e**) at days 1, 4, 8, and 12, (**f**) biodistribution of anti-miR-21 in different organs of animals 12 days after treatment with 3 consecutive doses (days 1, 4, and 8) [46].

In addition to drug encapsulation, inorganic nanoparticles themselves can serve as a means for cancer therapy. For example, MSC-derived exosomes loaded with 40 nm hollow Au nanoparticles allowed for direct monitoring of exosome trafficking between cells [48]. In particular, these exosomes only targeted the cell type of origin when comparing different cell types, such as MSCs, monocytes, and melanoma cells. These hollow Au nanoparticles exhibited near IR absorption (700–100 nm). Therefore, these hollow Au nanoparticle-loaded exosomes were responsive to NIR radiation and induced selective cell death through a photothermal effect. In another study, V_2_C QDs-loaded exosomes not only enabled PAI and MRI imaging, but also exhibited high effectiveness in photothermal therapy *in vitro* under near IR–II laser irradiation [30]. Significant apoptosis of MCF cells treated with V_2_C QDs-loaded exosomes was observed under 1064 nm laser irradiation of 0.96 W cm^−2^ power intensity for 10 min. This NIR laser irradiation led to a temperature increase up to 45 °C. The *in vivo* photothermal effects were also observed in tumor-bearing mice with intravenous injection of the V_2_C QDs-loaded exosomes. Specifically, animal groups with the injection of V_2_C QDs-loaded exosomes showed evident temperature increase at the tumor sites and significant tumor growth repression under laser irradiation (Figure 6a–c). The IR thermal images and temperature changes at the tumor sites clearly demonstrated the temperature increase after laser irradiation (10 min 1064 nm, 0.96 W cm^−2^) as shown in Figure 6a,b. Figure 6c shows the comparison of the tumor growth curves of the MCF-7 tumor-bearing mice in different groups, suggesting that the RGD and TAT peptide functionalization greatly enhanced the efficiency of the photothermal therapy [30]. In addition, no adverse side effects were observed, indicated by the minimal animal weight fluctuation and tissue damage.

Another multi-functional nanocarrier was created by loading mouse breast cancer cells-derived exosomes with Au-iron oxide hybrid nanoparticles and therapeutic agents (miRNA and DOX) [46]. This nanoplatform provided an excellent multimodal contrast agent for T_2_-weighted MR imaging, combined chemo-sensitizing miRNA, and photothermal effects. These exosomes exhibited improved tumor-specific targeting with minimal immune responses. *In vivo* biodistribution and anti-tumor properties of these tumor cell derived exosomes loaded with Au-iron oxide nanoparticles and anti-miR-21 with/without DOX showed distinct therapeutic effects (Figure 6d,e). This observation suggested that anti-miR-21 delivery provided additive effects to DOX, and reduced DOX resistance in breast cancer cells. The co-delivery of anti-miR-21 with DOX yielded a three-fold higher cell kill efficiency than using DOX alone. The quantification of anti-miR-21 suggested a preferable localization of those exosomes in 4_T1_ tumors as compared with other organs (Figure 6g).

Furthermore, iron oxide nanoparticles are able to generate heat under an alternating magnetic field (AMF), so-called magnetic hyperthermia. Iron oxide loaded exosomes derived from human MSCs exhibited tumor targeting and accumulation ability [49], where iron oxide nanoparticles were incorporated inside exosomes after those nanoparticles were endocytosis by MSCs. After secretion, those exosomes simultaneously loaded with iron oxide nanoparticles and mRNA. Those exosomes were easily taken up by tumor cells *in vitro* and caused cell death under AMF. Mouse MCS-derived exosomes loaded with citrate-coated iron oxide nanoparticles via biological pathways showed increased activation and migration ability of macrophage, suggesting that loading of iron oxide nanoparticles enhanced the immunoregulatory properties of the vesicles from MSCs [50]. All these studies suggested the great potential of inorganic nanoparticle-loaded exosomes in therapeutic applications.

### 3.3. Inorganic Nanoparticle-Loaded Exosomes for Other Applications

**Figure 7:**
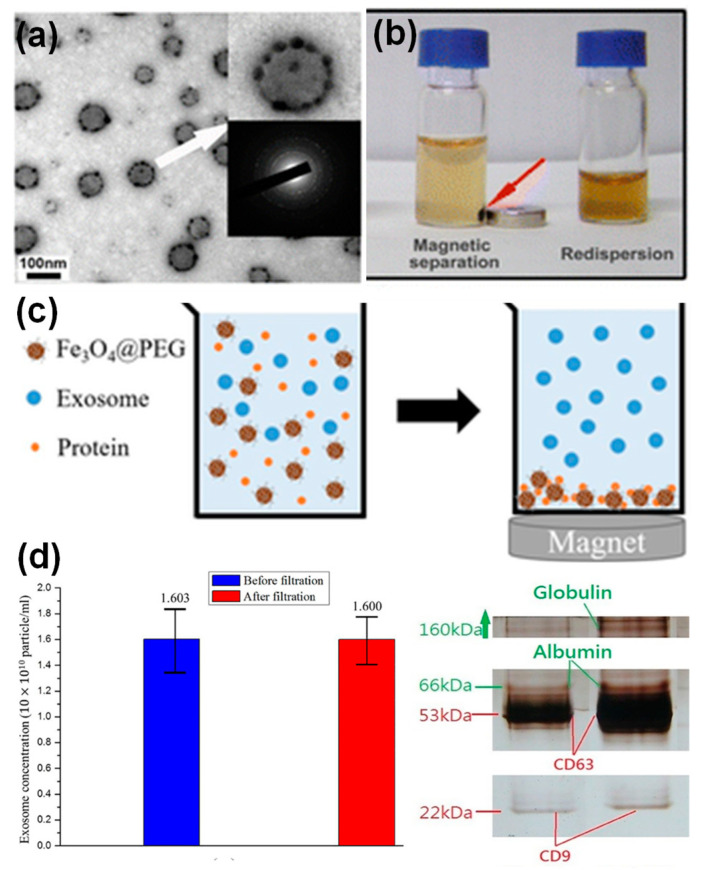
Iron oxide nanoparticles for exosome enrichment: a TEM image (**a**) and magnetic response (**b**) of Tf functionalized iron oxide decorated exosomes from blood serum [51]; PEG functionalized iron oxide nanoparticles for exosome purification: (**c**) principle and (**d**) exosome quantification before and after purification and SDS-PAGE gel of purified exosomes [20].

In addition to bioimaging and therapy, exosomes incorporated with nanoparticles, in particular, magnetic nanoparticles have been applied for enrichment and purification. For example, transferrin functionalized iron oxide nanoparticles have been linked to the surface Tf receptors of reticulocyte-derived exosomes from blood serum via Tf–Tf receptor interactions [51]. The decoration of multiple iron oxide nanoparticles on an exosome surface resulted in enhanced magnetic response of those exosomes to an external magnetic field. Subsequently, a significant increase in the exosome yield (~100 times) was obtained compared to exosome yields from cell cultures. Figure 7a,b showed the morphology of the iron oxide nanoparticle decorated exosomes, and their response to an external magnet. Alternatively, iron oxide nanoparticles have been applied to remove serum proteins via non-specific adsorption, leaving a cleaner exosome solution for further isolation and purification [20]. It is well known that nanoparticles with various surface coating nonspecifically adsorb serum proteins [52], which is normally considered a drawback of nanoparticles in drug delivery. In a recent study, the protein adsorption on nanoparticle surface was utilized in a positive way where PEG-coated iron oxide nanoparticles were applied for exosome purification [20]. Figure 7c shows the purification principle, which was tested using exosome solutions containing different protein supplements. The exosome concentration before and after nanoparticle treatment was not evidently altered, suggesting a minimal loss of exosomes (Figure 7d). SDS-PAGE gel of the purified exosomes demonstrated the effective removal of serum albumin and immunoglobulin proteins from solutions (Figure 7e). This study suggested that nanoparticles could offer another purification strategy for exosome preparation.

## 4. Summary and Outlook

In summary, we have discussed the preparation methods and emerging applications of inorganic nanoparticle-loaded exosomes. Among all the preparation methods, treating exosome-secreting cells with inorganic nanoparticles to include nanoparticles simultaneously during exosome formation is the most attractive. This method leads to minimal disruption of the surface protein markers of exomes and maintains the activities originated from parental cells, such as tumor targeting ability of tumor cell-derived exosomes. However, the loading efficiency for this method heavily depends on the size of the nanoparticles, which worked well for nanoparticles less than 10 nm, while larger size nanoparticles were more efficient to incorporate on the surface of the exosomes post exosome formation. In addition, the surface functionalities of the nanoparticles directly affects the cellular uptake and subsequent nanoparticle loading into exosomes, which is another factor to be considered when using this method. If the size of inorganic nanoparticles is small enough (<3 nm), electroporation could offer another effective method for nanoparticle loading into exosomes. Inorganic nanoparticle-loading exosomes have been primarily explored for tracking the exomes using various imaging modalities, such as iron oxide nanoparticle incorporation for MR imaging, Au nanoparticle loading for CT imaging, or QD encapsulation for fluorescence imaging. Among those imaging modalities, encapsulation of iron oxide nanoparticles for MR imaging is highly attractive because this technique is noninvasive and free of radiation. In particular, combination of imaging and therapy could open up potential new opportunities for cancer treatment. As for therapeutic effects, inorganic nanoparticles with NIR absorption have been shown to be an effective strategy for photothermal therapy because of the deep tissue penetration of NIR.

Even though numerous studies have been reported, it has been challenging to make any conclusive cross comparison because of the use of different cell lines, various types of nanoparticles, variations in the sizes and surface coating for the same type of nanoparticles, and discrepancies in data collection and analysis. Several technical hurdles remain prior to clinical translation of inorganic nanoparticle-loaded exosomes. For example, reproducible and standardized protocols are needed for the cross comparison among studies. In addition, systematic investigations on the effects of inorganic parameters on loading efficiency and subsequent applications need be performed. In this regard, the nanoparticle size, surface chemistry, and stability will not only affect the loading efficiency, but also define the ultimate fates. The ultimate fate of the nanoparticles is critical for long-term safety, which needs to be further investigated in comparing with free nanoparticles to understand whether the exosomes alter the inorganic nanoparticle fates. Furthermore, methodologies need to be developed to evaluate the association between exosomes and nanoparticles after *in vivo* administration. In particular, the time course for nanoparticle dissociation from exosomes is critical.

## Figures and Tables

**Table 1:** A summary of inorganic nanoparticle-loaded exosomes through physical methods.

Nanoparticles	Size (nm)	Exosome Origin	Preparation Method	Hybrid Structure	Ref.
QDs	10–20 nm	Body fluids	Surface Conjugation	Nanoparticles on exosome surfaces	[25]
Au	40 nm	MCF-7 cells	Surface Conjugation	Nanoparticles on exosome surfaces	[26]
Hollow Au	40 nm	B16-F10 cells	Electroporation (950 µF)	Multiple nanoparticle encapsulation	[27]
Au	20–30 nm	HEK293T cells	Extrusion	Single nanoparticle encapsulation	[28]
Iron Oxide	5 nm	B16-F10 cells	Electroporation with 50 mM trehalose	Multiple nanoparticle encapsulation	[29]
V_2_C QDs	16 nm	MCF-7 cells	Electroporation (200 V, 100 µF)	Multiple nanoparticle encapsulation	[30]
Ag_2_Se@Mn	2 nm	MCF-7, CAL27, and A549 cells	Electroporation (250 V, 350 µF)	Multiple nanoparticle encapsulation	[31]
Au	5 and 20 nm	Human MSCs	Passive incubation (37 °C, 3 h)	N/A	[32]

## Data Availability

The data presented in this study are openly available in Molecules 2021, 26, 1135. http://doi.org/10.3390/molecules26041135.
